# Melatonin suppresses ethylene biosynthesis by inhibiting transcription factor MdREM10 during apple fruit ripening

**DOI:** 10.1093/hr/uhaf020

**Published:** 2025-01-21

**Authors:** Chen Li, Qian Yu, Yajing Si, Yuling Liang, Shijiao Lin, Guangxin Yang, Weiting Liu, Yinglin Ji, Aide Wang

**Affiliations:** Key Laboratory of Fruit Postharvest Biology (Liaoning Province), College of Horticulture, Shenyang Agricultural University, No. 120, Dongling Road, Maganqiao Street, Shenhe District, Shenyang 110866, China; Key Laboratory of Fruit Storage and Processing (Liaoning Province), Chinese Academy of Agricultural Sciences Research Institute of Pomology, No. 98, Xinghai South Street, Wenquan Street, Xingcheng 125100, China; Key Laboratory of Fruit Postharvest Biology (Liaoning Province), College of Horticulture, Shenyang Agricultural University, No. 120, Dongling Road, Maganqiao Street, Shenhe District, Shenyang 110866, China; Key Laboratory of Fruit Postharvest Biology (Liaoning Province), College of Horticulture, Shenyang Agricultural University, No. 120, Dongling Road, Maganqiao Street, Shenhe District, Shenyang 110866, China; Key Laboratory of Fruit Postharvest Biology (Liaoning Province), College of Horticulture, Shenyang Agricultural University, No. 120, Dongling Road, Maganqiao Street, Shenhe District, Shenyang 110866, China; Key Laboratory of Fruit Postharvest Biology (Liaoning Province), College of Horticulture, Shenyang Agricultural University, No. 120, Dongling Road, Maganqiao Street, Shenhe District, Shenyang 110866, China; Key Laboratory of Fruit Postharvest Biology (Liaoning Province), College of Horticulture, Shenyang Agricultural University, No. 120, Dongling Road, Maganqiao Street, Shenhe District, Shenyang 110866, China; Key Laboratory of Fruit Postharvest Biology (Liaoning Province), College of Horticulture, Shenyang Agricultural University, No. 120, Dongling Road, Maganqiao Street, Shenhe District, Shenyang 110866, China; Key Laboratory of Fruit Postharvest Biology (Liaoning Province), College of Horticulture, Shenyang Agricultural University, No. 120, Dongling Road, Maganqiao Street, Shenhe District, Shenyang 110866, China

## Abstract

Ethylene, a plant hormone, is essential for apple (*Malus domestica*) ripening. The precise molecular mechanism by which melatonin (MT) influences ethylene biosynthesis during apple fruit ripening remains unclear. This study found that exogenous MT treatment inhibited ethylene production and postponed apple fruit ripening. The endogenous MT content of apple fruits exhibited an inverse correlation with ethylene production during fruit ripening, suggesting that MT functions as a ripening suppressor in apple fruits. MT treatment suppressed the expression of key ethylene biosynthesis genes, *MdACS1* and *MdACO1*, during apple fruit ripening. MT treatment decreased the expression levels of transcription factors *MdREM10* and *MdZF32*. MdREM10 binds to the *MdERF3* promoter, enhancing its expression and subsequently promoting *MdACS1* transcription. Furthermore, MdREM10 directly bound to the *MdZF32* promoter, promoting its transcription. MdZF32 directly bound to the *MdACO1* promoter, inducing its expression. The findings suggested that MT suppresses ethylene biosynthesis and fruit ripening by inhibiting MdREM10, which indirectly promotes *MdACS1* transcription via *MdERF3* upregulation, and *MdACO1* transcription via *MdZF32* upregulation.

## Introduction

Apple (*Malus domestica*), a globally prevalent fruit crop, significantly contributes to human nutrition by supplying essential nutrients [1]. The storage properties of apples are of significant economic importance, and improper storage techniques cause considerable losses in crop value, which depend on fruit ripening [2]. Ripening is a key physiological process regulated by gene transcription during fruit development. It encompasses various metabolic alterations influencing the fruits’ color, flavor, texture, and aroma [3, 4]. These changes are influenced by endogenous hormones, genetic regulators, transcription factors (TFs), and external signals like temperature, light, and hydration [3]. Apple, as a typical climacteric fruit, is regulated by ethylene [5]. Lowering ethylene levels delays softening and extends the shelf life of climacteric fruit [6].

In the ethylene biosynthesis pathway, ACC synthase (ACS) transforms *S*-adenosylmethionine (SAM) to 1-aminocyclopropane-1-carboxylic acid (ACC), which ACC oxidase (ACO) then oxidizes to produce ethylene [7]. Silencing of *ACS* and *ACO* in apples has been demonstrated to block ethylene biosynthesis and prolong shelf life [8, 9]. In ethylene signal transduction, the ethylene receptor activates the pathway involving CONSTITUTIVE TRIPLE RESPONSE1 (CTR1) and Ethylene INSENSITIVE2 (EIN2). The signal is then transmitted to the primary TF EIN3/EIN3-like, which controls the secondary transcription of ETHYLENE RESPONSE FACTOR (ERF). The expression of downstream ethylene response genes is controlled by ERFs [10, 11]. MaERF11 binds to the promoters of *MaACS1* and *MaACO1*, resulting in their repression in bananas (*Musa acuminata*) [12]. In apples, MdERF2 and MdERF3, respectively, suppresses and promotes *MdACS1* transcription [13], and MdERF5 suppresses *MdACS1* transcription [14]. Ethylene synthesis is regulated by diverse TF families, including NAC, Dof, HSF, MYB, HD-Zip homeobox protein, and MADS-box proteins [15–20]. In kiwifruits (*Actinidia deliciosa*), AdNAC6 and AdNAC7 promote *AdACS1* and *AdACO1* transcription and increase ethylene production [19]. In tomatoes (*Solanum lycopersicum*), the HD-Zip homeobox protein SlHB-1 binds to the *SlACO1* promoter, promoting its activity in regulating fruit ripening [21]. In grapes (*Vitis vinifera*), both VvMYB108A and VvMYB14 increase ethylene production by activating *VvACS1* transcription [22, 23]. In apples, the transcriptional activity of the *MdACS1* promoter is enhanced by MdHSFB2a, MdDof1.2, and the MADS-box TF MdAGL104, whereas it is inhibited by MdAGL30, MdERF008, MdNAC71, and MdHSFB3 [20].

Additional plant hormones affect ethylene biosynthesis during ripening. The exogenous synthetic auxin naphthalene acetic acid (NAA) promotes *MdACS1* and *MdACO1* transcription, inducing ethylene biosynthesis in apples through the activation of the TF MdARF5-mediated auxin signaling [24]. Jasmonate promotes *MdACS1* and *MdACO1* transcription by activating the expression of *MdMYC2*, facilitating ethylene biosynthesis in apples [25]. Abscisic acid (ABA) treatment induces ethylene production by upregulating *SlACS* and *SlACO* expression in tomatoes [26]. Brassinolides (BRs) have been demonstrated to suppress the transcription of *PuERF2*, *PuACO1*, and *PuACS1a* by upregulating the expression of *PuBZR1*, inhibiting ethylene biosynthesis in pear (*Pyrus ussuriensis*) fruits [27]. Nevertheless, the precise mechanism by which melatonin (MT) affects ethylene biosynthesis remains unclear. MT is found in various plants and all their organs, including leaves, roots, flowers, and fruits, at various concentrations [28–30]. It is involved in various plant physiological processes such as seed germination, root development, photosynthesis, and senescence [31, 32]. It contributes to the process of fruit ripening [33]. For example, studies have demonstrated that ABA and MT interact to affect physiological processes such as fruit tolerance and ripening [34–36]. MT levels have been observed to vary with fruit development in different plant species. MT levels increase during the growth phase and subsequently decline during ripening in both grapes and tomatoes [34, 37]. In cherries (*Prunus avium*), the application of 100 μM MT to trees before harvest resulted in a delay in fruit ripening [35]. MT has been shown to delay postharvest aging in several fruit species, including pears (*Pyrus communis*) [38] and apples [39].

The aforementioned studies indicate that MT exerts an influence on ethylene biosynthesis. However, the molecular mechanism by which MT influences ethylene biosynthesis during ripening remains unknown, and the involved TFs have not yet been identified. This study showed that MT suppresses the expression of the transcription activator *REPRODUCTIVE MERISTEM 10* (*MdREM10*), leading to the upregulating of genes associated with ethylene biosynthesis. This leads to a reduction in ethylene production and suppression of fruit ripening.

## Results

### MT acts as a suppressor for ethylene biosynthesis of apple fruits

**Figure 1:**
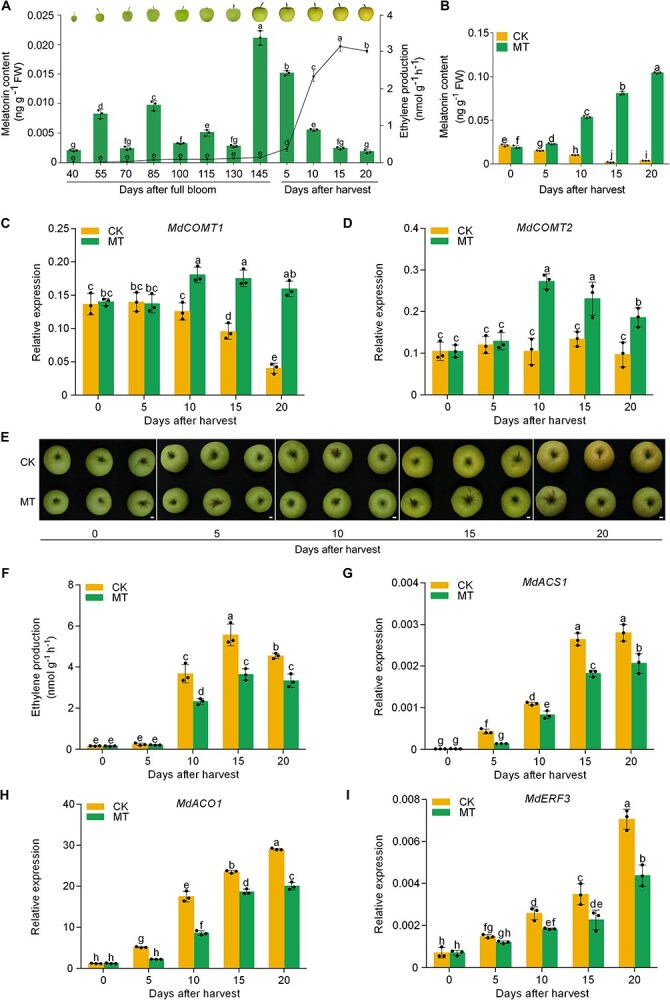
**MT treatment suppresses apple fruit ripening**. (A) The MT content and ethylene production in apple fruits during 2022. The bar chart shows MT content and the line chart shows ethylene production. (B) MT content was determined after treatment. (C, D) RT-qPCR was used to assess the expression levels of *MdCOMT1* and *MdCOMT2* throughout fruit ripening. (E) Apple fruits were collected 140 days post-full bloom (commercial harvest). MT, the apple fruits were treated with MT. CK, the apple fruits without treatment as the controls. (F) Ethylene production was assessed following the treatment. (G–I) RT-qPCR was employed to measure the expression levels of *MdACS1*, *MdACO1*, and *MdERF3* during fruit ripening. Data represent the means ± SE. Distinct lowercase letters denote significant differences at *P <* 0.05, determined by a one-way ANOVA test for A and a two-way ANOVA test for B–D and F–I.

In 2021, the endogenous MT levels in ‘Golden Delicious’ (GD) apples were evaluated throughout their development and ripening stages (Fig. S1) and in 2022 (Fig. 1A) to clarify the function of MT in the process of fruit ripening. Endogenous MT content increased markedly 140 days after full bloom (DAFB, commercial harvest) and then gradually decreased. This contrasted with the ethylene production profile observed during fruit ripening (Fig. 1A), indicating a negative correlation between MT content and ethylene production. In 2021, GD fruits harvested at 140 DAFB were treated with MT concentrations of 0.01, 0.1, 0.5, 1, and 2 mM. The low-MT concentration treatments (0.01 and 0.1 mM) showed no significant difference to the control fruits. Ethylene production was significantly inhibited by 0.5-, 1-, and 2-mM treatments compared to the untreated control fruits. The 0.5 mM MT treatment demonstrated the most pronounced effect (Fig. S2) and was therefore selected for further investigation. Following MT treatment, both the MT content and the transcription levels of MT biosynthesis genes *MdCOMT1* and *MdCOMT2* [40] in the fruits showed a significant increase (Fig. 1B–D). MT treatment similarly influenced ethylene production and fruit ripening by 2022 (Fig. 1E and F). Furthermore, we treated GD fruits with ethephon and subsequently measured the endogenous MT content. The application of ethephon did not result in any discernible impact on the endogenous MT content (Fig. S3). These findings suggested that MT functions as an ethylene inhibitor.

To understand how MT inhibits ethylene biosynthesis, we measured the transcription levels of key ethylene biosynthesis genes, *MdACS1* and *MdACO1*, in apples. Our study demonstrated that MT treatment markedly inhibits the transcription of *MdACS1* and *MdACO1* (Fig. 1G and H). ERFs play a crucial role in the ethylene signaling pathway. To understand the role of ERFs in apple ripening, the expression of four associated ERFs (*MdERF2*, *MdERF3*, *MdERF4*, and *MdERF5*) was analyzed [13, 14, 41]. MT treatment significantly suppressed *MdERF3* expression (Fig. 1I). The expression of *MdERF2*, *4*, and *5* in apple fruits was not affected by MT treatment (Fig. S4). These findings suggested that MT reduces ethylene production in apples by inhibiting the expression of ethylene biosynthesis genes.

### MT inhibits MdREM10 to suppress ethylene biosynthesis

**Figure 2:**
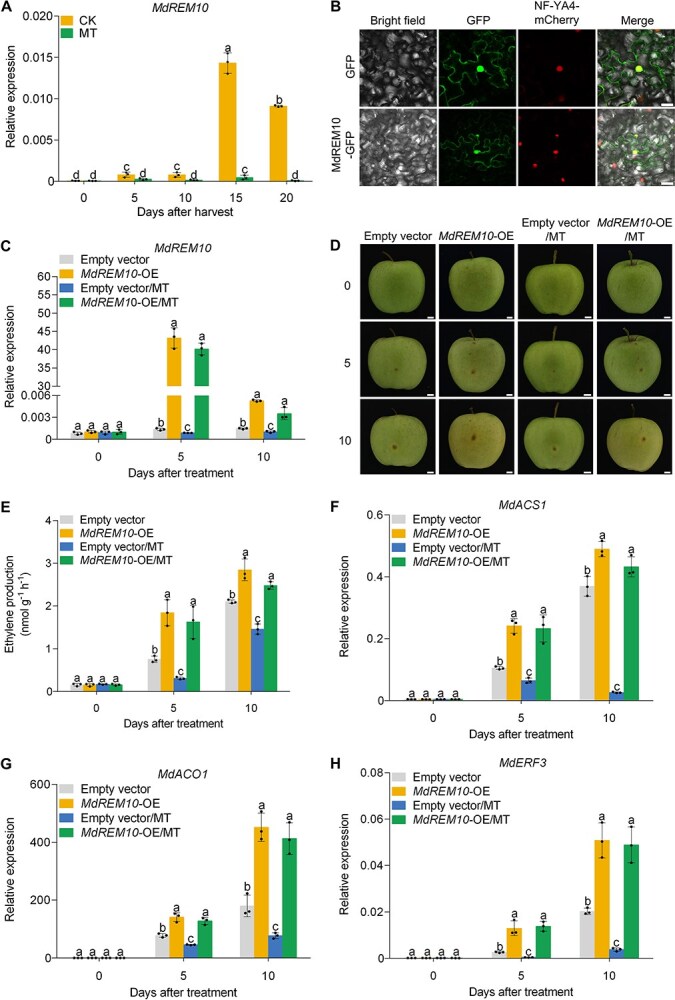
**MdREM10 is essential for the suppression of ethylene biosynthesis by MT in apple fruits**. (A) Expression of *MdREM10* in apple fruits treated with MT. (B) Subcellular localization of MdREM10. MdREM10-GFP was transformed into *N. benthamiana* leaves. Transient GFP expression was used as the control. The mCherry-labeled NF-YA4-mCherry served as a nuclear marker. Scale bars, 50 *μ*m. (C) *MdREM10* expression in infiltrated fruits were assessed using RT-qPCR. (D) Transient overexpression of *MdREM10* (*MdREM10*-OE) in apple fruits via *Agrobacterium* at 140 days post-full bloom. Twenty-four hours postinjection, samples were stored at room temperature for 10 days. Fruits infiltrated with empty pRI101-GFP vectors were used as controls. At 0, 5, 10, days after MT treatment. Scale bars, 1 cm. (E) Ethylene production in infiltrated fruits was determined. (F–H) RT-qPCR evaluated the expressions of *MdACS1*, *MdACO1*, and *MdERF3* in infiltrated fruits. Data represent the means ± SE. Distinct lowercase letters denote significant differences at *P <* 0.05, determined by a two-way ANOVA test for A and a one-way ANOVA test for C, E–H.

To clarify the role of MT-mediated TFs in regulating gene expression related to ethylene biosynthesis in apples, we performed a comparative transcriptomic analysis using RNA sequencing (RNA-seq) on GD fruits stored at room temperature (25°C) for 10 days, with and without MT treatment (Table S1). The expression of a TF with a B3 domain was notably reduced in fruits treated with MT (Table S2). The gene was subsequently cloned from GD fruits and designated MdREM10. We conducted reverse transcription-quantitative polymerase chain reaction (RT-qPCR) on fruit samples collected at 140 DAFB to assess the impact of MdREM10 on fruit ripening in response to MT. The results demonstrated that *MdREM10* expression parallels to that of ethylene, and is significantly suppressed by MT treatment (Fig. 2A). We then examined the subcellular localization of MdREM10. The *MdREM10* coding sequence (CDS) was fused to a green fluorescent protein (GFP) tag under the *35S* promoter (MdREM10-GFP) and introduced into *Nicotiana benthamiana* leaves. Microscopic analysis identified MdREM10 in both the cytoplasm and nucleus (Fig. 2B).

We transiently overexpressed *MdREM10* (*MdREM10*-OE) in apples to study its role in ethylene biosynthesis. The overexpression construct MdREM10-GFP was infiltrated into GD fruits harvested 140 DAFB. Fruits containing the empty pRI101-GFP vector were used as controls. Fruits were infiltrated with 0.5 mM MT and stored at room temperature for 10 days. We examined the mRNA and DNA levels and observed that *MdREM10* was successfully overexpressed in apple fruits (Fig. 2C; Fig. S5A). We observed that *MdREM10*-OE fruits ripen faster than the control fruits and that MT treatment does not significantly suppress the ripening of *MdREM10*-OE fruits (Fig. 2D). Ethylene production in *MdREM10*-OE fruits exceeded that of control fruits and was not significantly reduced by MT treatment (Fig. 2E). The expression levels of *MdACS1*, *MdACO1*, and *MdERF3* were significantly higher in *MdREM10*-OE fruits compared to control fruits, even after MT treatment (Fig. 2F–H). These results suggested that MdREM10 is important for MT to suppress ethylene production in apples.

### MT restrains MdREM10 from activating the expression of *MdERF3*

**Figure 3:**
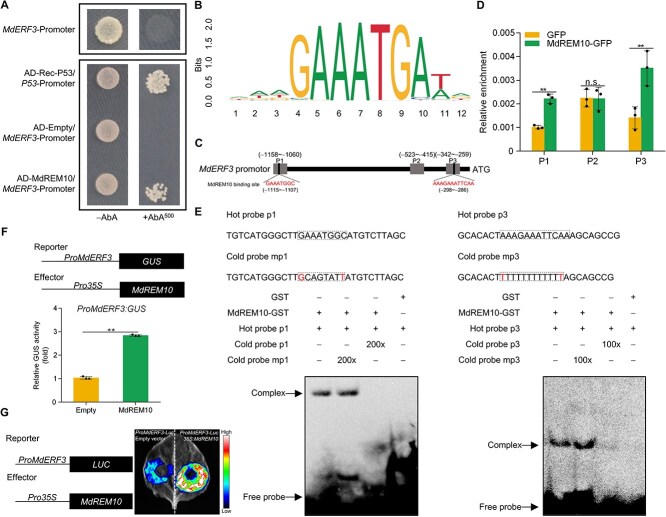
**MdREM10 promotes transcription of *MdERF3*** *.* (A) Y1H analysis demonstrated that MdREM10 binds to the *MdERF3* promoter. AD-Rec-P53/*P53*-Promoter served as a positive control. The AD-Empty/*MdERF3*-Promoter was used as a negative control. (B) The JASPAR database (https://jaspar.genereg.net/) predicts binding motifs for MdREM10. (C) A schematic of potential MdREM10-binding sites in the *MdERF3* promoter region. (D) ChIP-PCR analysis revealed that MdREM10 binds to the *MdERF3* promoter *in vivo*. Cross-linked chromatin samples were extracted from apple fruits overexpressing MdREM10-GFP and precipitated with anti-GFP antibodies. The eluted DNA was amplified for adjacent binding motif sequences using qPCR. Three segments (P1, P2, and P3) were investigated. Fruits with GFP overexpression were used as negative controls. The ChIP test was conducted thrice, with each enriched DNA fragment serving as a biological replicate for the qPCR. (E) EMSA demonstrated that MdREM10 interacts with the *MdERF3* promoter. The hot probe is a biotin-labeled fragment with the MdREM10-binding motif in the *MdERF3* promoter. The cold probe is an unlabeled competitive probe present at 100 and 200 times the concentration of the hot probes, while the mutant probe is an unlabeled cold probe sequence with a mutated binding motif. Purified GST-labeled MdREM10 was used, with GST protein serving as a negative control. (F) GUS activation assay showed that MdREM10 activates the *MdERF3* promoter (*ProMdERF3*). Three transfection experiments were independently conducted. (G) The dual-luciferase reporter assay demonstrates that MdREM10 enhances *MdERF3* promoter activity. Data represent the means ± SE. Asterisks denote significant differences (^**^P < 0.01, Student’s *t*-test). n.s., no significant difference.

The overexpression of *MdREM10* in apple fruits increased the expression of *MdACS1*, *MdACO1*, and *MdERF3* (Fig. 2F–H). Based on these findings, we hypothesized that MdREM10 regulates the transcription of these genes. A yeast one-hybrid (Y1H) assay revealed that MdREM10 bound to the *MdERF3* promoter (Fig. 3A), but not to the *MdACS1* or *MdACO1* promoter (Fig. S6). To ascertain whether MdREM10 is involved in ethylene synthesis through the direct regulation of *MdERF3* transcription, we employed the JASPAR database (https://jaspar.genereg.net/) to predict MdREM10-binding sites within the *MdERF3* promoter region, identifying two putative motifs with high probability (AAA(T/G)(G/T)) (Fig. 3B and C). We performed a chromatin immunoprecipitation (ChIP)-PCR experiment to verify binding *in vivo*. The *MdREM10* CDS was cloned upstream of the GFP tag, driven by the *35S* promoter in the pRI101 vector, and expressed in apples. The ChIP-PCR results indicated that MdREM10 was enriched in P1 and P3 fragments of the *MdERF3* promoter (Fig. 3C and D). An electrophoretic mobility shift assay (EMSA) was conducted to assess the direct interaction between MdREM10 and specific motifs within the *MdERF3* promoter. Biotin-labeled, unlabeled, and mutated probes were synthesized based on the predicted binding sites illustrated in Fig. 3E. The results demonstrated that MdREM10 exhibited binding activity to the binding motifs (P1 and P3 fragments) within the *MdERF3* promoter. A *β*-Glucuronidase (GUS) activation assay was performed to investigate the regulation of MdREM10 on the *MdERF3* promoter in *N. benthamiana* leaves. The cotransformation of the *Pro35S:MdREM10* and *ProMdERF3:GUS* constructs demonstrated an increase in GUS activity compared to the empty pRI101 vector and *ProMdERF3:GUS* constructs, indicating that MdREM10 activates the *MdERF3* promoter (Fig. 3F). Dual-luciferase reporter assays also yielded results consistent with those of GUS activation assays (Fig. 3G). As reported by Li *et al.* [13], MdERF3 enhances the expression of *MdACS1* by interacting with its promoter. These results indicated that MT prevents MdREM10 from indirectly activating *MdACS1* transcription via MdERF3.

### MT restrains MdZF32 from activating the expression of *MdACO1*

**Figure 4:**
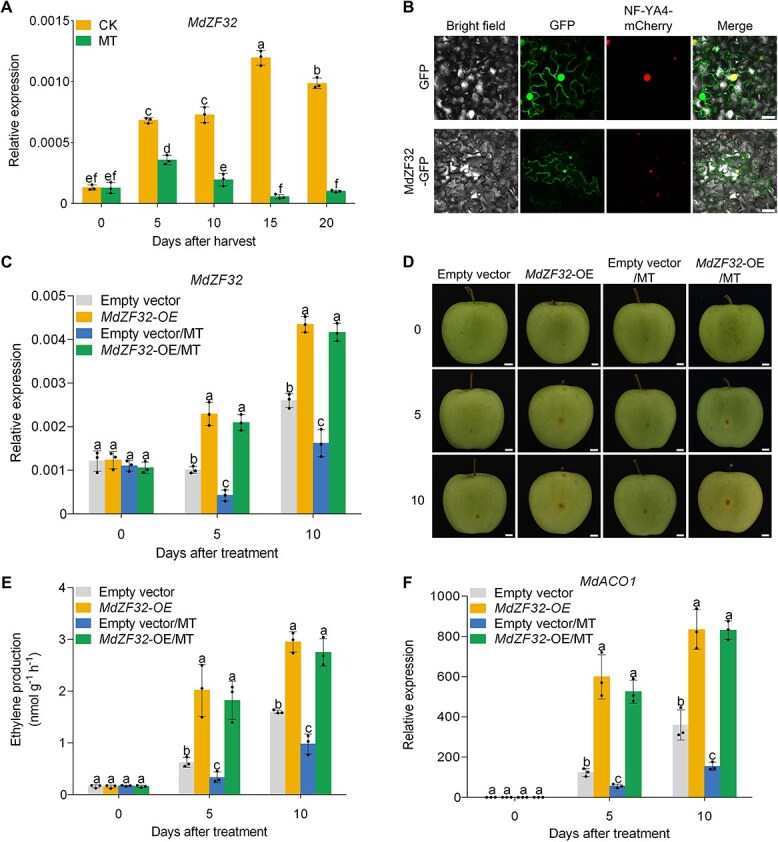
**MdZF32 plays a role in the suppression of ethylene biosynthesis by MT in apple fruits**. (A) Expression of *MdZF32* in apple fruits treated with MT. Three biological replicates of independent RNA extraction from each group of fruits were analyzed. (B) Subcellular localization of MdZF32. MdZF32-GFP was transformed into *N. benthamiana* leaves. Transient GFP expression was used as the control. The mCherry-labeled NF-YA4-mCherry served as a nuclear marker. Scale bars, 50 *μ*m. (C) *MdZF32* expression in infiltrated fruits was assessed using RT-qPCR. (D) Transient overexpression of *MdZF32* (*MdZF32*-OE) in apple fruits using *Agrobacterium*-mediated methods, 140 days post-full bloom. 24 h after MT injection, samples were stored at room temperature for 10 days. Fruits infiltrated with the empty pRI101-GFP vector served as controls. At 0, 5, 10, days after MT treatment. Scale bars, 1 cm. (E) Ethylene production in infiltrated fruits was determined. (F) RT-qPCR assessed *MdACO1* expression in infiltrated fruits. Data represent the means ± SE. Distinct lowercase letters denote significant differences at *P <* 0.05, as determined by a two-way ANOVA test for A and a one-way ANOVA test for C, E, and F.

MT treatment notably reduced the expression of ethylene biosynthesis genes *MdACS1* and *MdACO1* (Fig. 1G and H). Qiu *et al.* [42] reported that overexpression of the zinc finger protein (ZFP) *CaC3H14* leads to downregulation of *CaACO1* expression in capsicum (*Capsicum annuum*). We identified four differentially expressed ZFP genes (MD03G1279200, MD03G1283900, MD17G1235500, and MD03G1280300) via RNA-seq (Table S2) and confirmed their expression in 140-DAFB fruits using RT-qPCR to assess their potential role in fruit ripening. We observed that only the expression of MD17G1235500 was parallel to ethylene production and was decreased by MT treatment during ripening (Fig. 4A; Fig. S7). Based on sequence alignment, it was named *MdZF32*. We examined the subcellular localization of MdZF32 and found it presented in both the cytoplasm and nucleus (Fig. 4B).

To investigate the role of MdZF32 in ethylene production, we fused the CDS of *MdZF32* to GFP tags under the *35S* promoter and transiently overexpressed it in apples harvested at 140 DAFB (*MdZF32*-OE). Fruits infiltrated with the empty pRI101-GFP vector served as controls. The expression of *MdZF32* in *MdZF32*-OE fruits was significantly higher than that in the control fruits. Fruits treated with 0.5 mM MT and stored at room temperature for 10 days exhibited significantly higher *MdZF32* expression in *MdZF32*-OE fruits compared to control fruits (Fig. 4C). We also examined the DNA level and observed that *MdZF32* was successfully overexpressed in apple fruits (Fig. S5B). *MdZF32*-OE fruits ripened faster than control fruits, and MT treatment did not significantly suppress the ripening of *MdZF32*-OE fruits (Fig. 4D). Ethylene production was higher in *MdZF32*-OE fruits compared to control fruits (Fig. 4E). MT treatment significantly suppressed ethylene production in control fruits but had no effect on *MdZF32*-OE fruits (Fig. 4E), similar to *MdACO1* expression (Fig. 4F). However, the expression levels of *MdACS1* and *MdERF3* did not differ between the *MdZF32*-OE and the control fruits (Fig. S8A and B). These findings suggested that MdZF32-mediated MT may suppress ethylene production in apples by regulating *MdACO1*.

**Figure 5:**
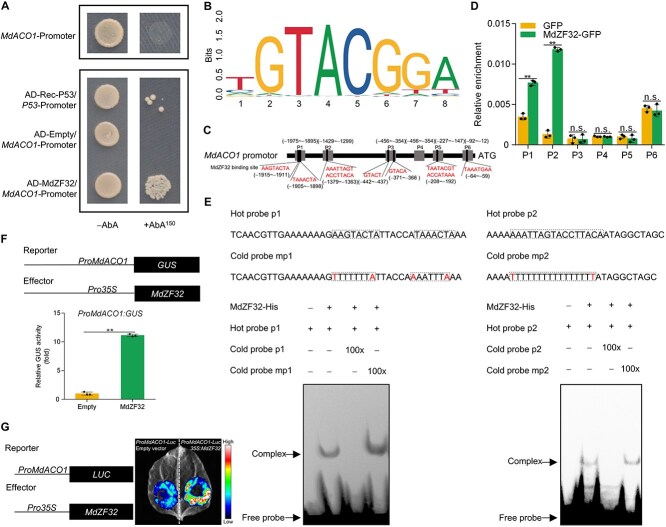
**MdZF32 promotes transcription of *MdACO1.*** (A) Y1H analysis demonstrated that MdZF32 binds to the *MdACO1* promoter. See Fig. 3A for additional details. (B) The JASPAR database (https://jaspar.genereg.net/) predicts binding motifs for MdREM10. (C) A schematic of potential MdZF32-binding sites in the *MdACO1* promoter region. (D) ChIP-PCR revealed that MdZF32 binds to the *MdACO1* promoter *in vivo*. See Fig. 3D for additional details. Six segments (P1, P2, P3, P4, P5, and P6) were investigated. Fruits with GFP overexpression were used as negative controls. (E) EMSA revealed *in vitro* binding of MdZF32 and *MdACO1* promoter. Purified poly-histidine (His)-labeled MdZF32. See Fig. 3E for additional details. (F) GUS activation assay showed that MdZF32 activates the *MdACO1* promoter (*ProMdACO1*). Three transfection experiments were independently conducted. (G) The dual-luciferase reporter assay demonstrates that MdZF32 modulates *MdACO1* promoter activity. Data represent the means ± SE. Asterisks denote significant differences (^**^P < 0.01, Student’s *t*-test). n.s., no significant difference.

A Y1H assay revealed that MdZF32 bound to the *MdACO1* promoter, suggesting its role in regulating *MdACO1* transcription and ethylene synthesis in apple fruits (Fig. 5A). We identified seven presumed motifs bound to MdZF32 with high probability in the *MdACO1* promoter using the JASPAR website (Fig. 5B and C) and performed ChIP-PCR to confirm whether MdZF32 bound to these motifs of the *MdACO1* promoter. The results showed that MdZF32 enriched only the P1 (−1975 to −1895 bp) and P2 (−1429 to −1299 bp) fragments of the *MdACO1* promoter (Fig. 5D). An EMSA was performed to identify whether MdZF32 directly binds to these specific binding motifs in the P1 and P2 fragments of the *MdACO1* promoter. Biotin-labeled, unlabeled, and mutated probes were designed and synthesized based on the predicted binding sites (Fig. 5E). The results showed that MdZF32 directly binds to the *MdACO1* promoter. Cotransformation with *Pro35S:MdZF32* and *ProMdACO1:GUS* constructs exhibited higher GUS activity than the empty pRI101 vector and *ProMdACO1:GUS* constructs, indicating MdZF32 activation of the *MdACO1* promoter (Fig. 5F). The dual-luciferase reporter assays also showed the same results with GUS activation assays (Fig. 5G). These findings indicated that MT prevents *MdACO1* transcription by suppressing *MdZF32* expression.

### MdREM10 acts upstream of *MdZF32* to regulate ethylene biosynthesis

**Figure 6:**
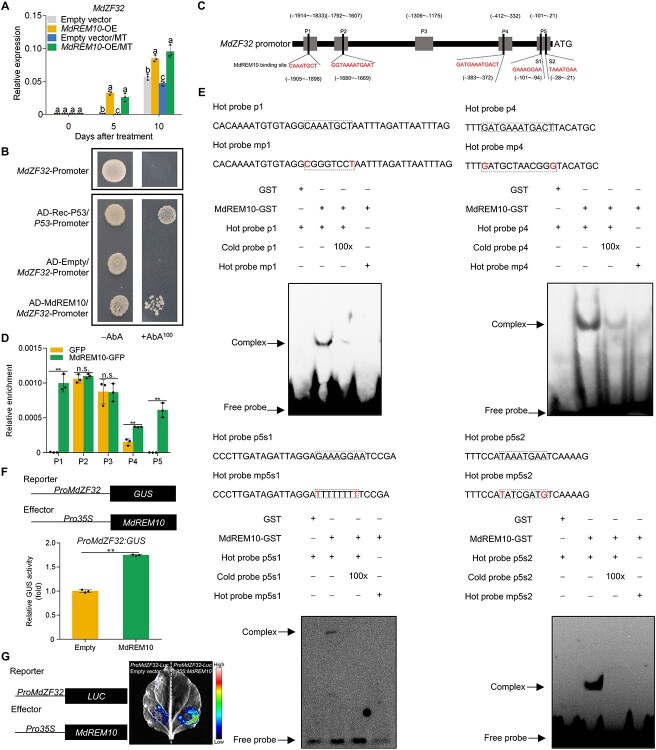
**MdREM10 promotes transcription of *MdZF32.*** (A) *MdZF32* expression in fruits overexpressed *MdREM10* (*MdREM10*-OE) were evaluated using RT-qPCR. Distinct lowercase letters denote a significant difference at *P <* .05, as determined by a one-way ANOVA test. (B) Y1H analysis demonstrated that MdREM10 binds to the *MdZF32* promoter. See Fig. 3A for additional details. (C) A schematic of potential MdREM10-binding sites in the *MdZF32* promoter region. (D) ChIP-PCR revealed that MdREM10 binds to the *MdZF32* promoter *in vivo*. See Fig. 3D for additional details. (E) EMSA revealed *in vitro* binding of MdREM10 to the *MdZF32* promoter. See Fig. 3E for additional details. (F) GUS activation assay showed that MdREM10 activates the *MdZF32* promoter (*ProMdZF32*). Three transfection experiments were independently conducted. (G) Dual-luciferase reporter assay demonstrates that MdREM10 modulates *MdZF32* promoter activity. Data represent the means ± SE. Asterisks denote significant differences (^**^P < 0.01, Student’s *t*-test). n.s., no significant difference.

To further explore the relationship among MdREM10, MdZF32, and MdERF3, we first performed a yeast two-hybrid assay (Y2H) and found that none of them interacted with each other (Fig. S9). Interestingly, we found that *MdZF32* expression is significantly higher in *MdREM10*-OE fruits compared to the control fruits (Fig. 6A), while *MdREM10* expression has no difference between the *MdZF32*-OE and the control fruits (Fig. S8C). These results suggested that MdREM10 acts upstream of *MdZF32* to regulate its transcription. A Y1H assay demonstrated that MdREM10 bound to the *MdZF32* promoter (Fig. 6B), while MdZF32 did not bind to the *MdREM10* promoter (Fig. S10). The four presumed motifs bound to MdREM10 with a high probability were predicted in the *MdZF32* promoter using the JASPAR website (Figs 3B and 6C). We found that MdREM10 was recruited to the P1 (−1914 to −1833 bp), P4 (−412 to −332 bp), and P5 (−109 to −9 bp) fragments of the *MdZF32* promoter by ChIP-PCR (Fig. 6D). An EMSA was performed to determine whether MdREM10 directly binds to these specific motifs in the P1, P4, and P5 fragments of the *MdZF32* promoter. Biotin-labeled, unlabeled, and mutated probes were designed and synthesized based on the predicted TF-binding sites (Fig. 6E). The results showed that MdREM10 directly binds to the *MdZF32* promoter. Cotransformation with *Pro35S:MdREM10* and *ProMdZF32:GUS* constructs demonstrated higher GUS activity than the empty pRI101 vector and *ProMdZF32:GUS*, indicating that MdREM10 activates the activity of *MdZF32* promoter (Fig. 6F). Dual-luciferase reporter assays yielded results consistent with those of GUS activation assays (Fig. 6G). These results suggested that MdREM10 acts upstream of *MdZF32* and MT prevents MdREM10 from indirectly activating *MdACO1* transcription via MdZF32.

## Discussion

**Figure 7:**
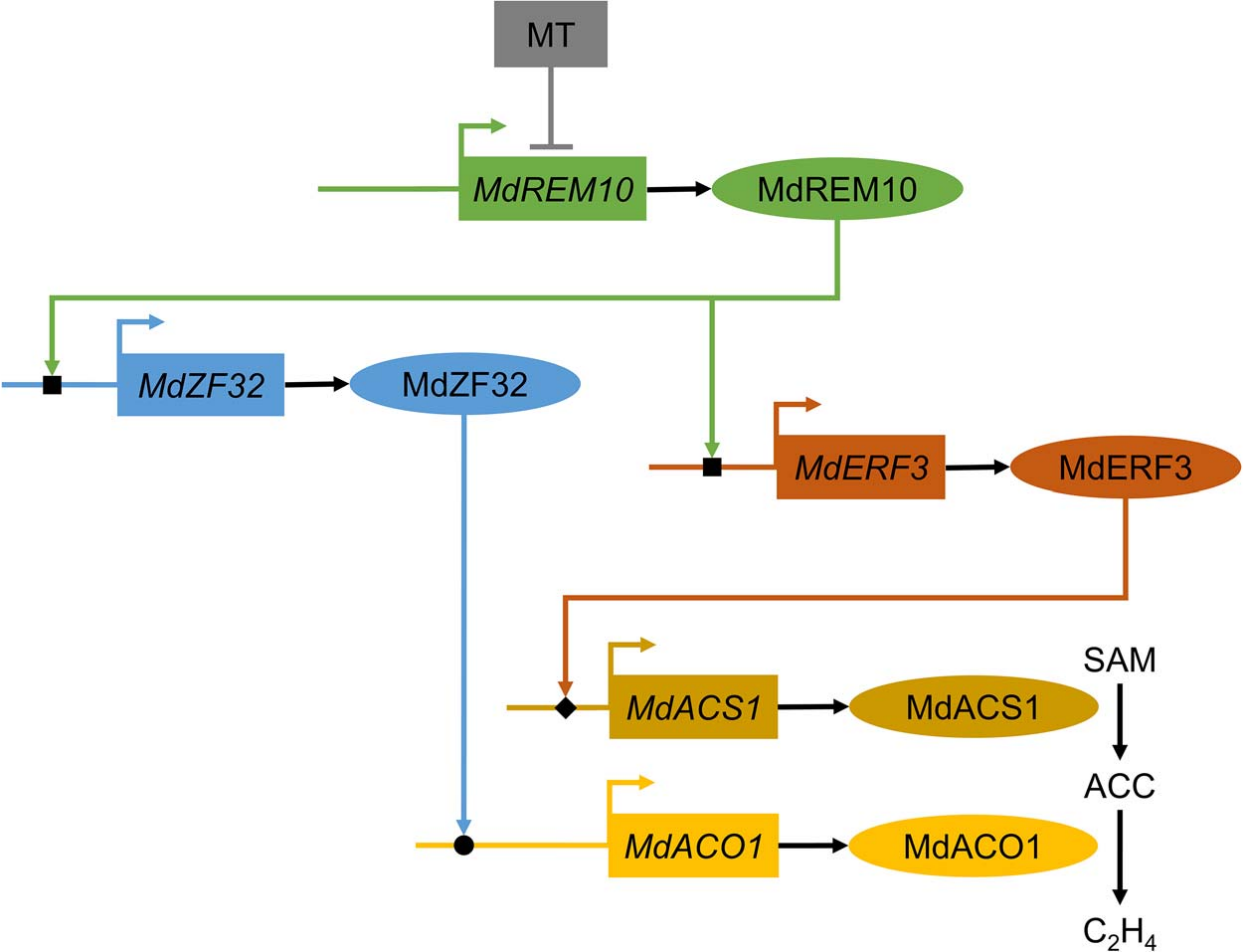
**A model showing the regulating of MT on apple fruit ripening.** During apple fruit ripening, MT suppresses *MdREM10* transcription. On the one hand, MT prevents MdREM10 from promoting *MdERF3* transcription, in which MdERF3 promotes *MdACS1* transcription; on the other hand, MT prevents MdREM10 from promoting *MdZF32* transcription, in which MdZF32 activates *MdACO1* transcription, suppressing ethylene biosynthesis and delaying ripening. ■, MdREM10-binding site; ●, MdZF32-binding site; ♦, MdERF3-binding site. Promotion is represented by a colored arrow, while suppression is shown by an up tack symbol. SAM refers to *S*-adenosyl methionine, ACC to aminocyclopropane-1-carboxylic acid, and C_2_H_4_ to ethylene.

MT, a novel class of hormone, is crucial in the plant life cycle. It regulates hormone balance by interacting with other plant hormones, including auxin (IAA), jasmonic acid (JA), and abscisic acid (ABA). The impact of MT on plant growth and stress responses is well documented [43, 44]. Previous studies have demonstrated that exogenous MT inhibits ethylene synthesis in bananas, pears, and apples [39, 45, 46]. However, the precise nature of the interaction between ethylene and MT during fruit ripening remains unclear. This study revealed that ethephon treatment does not affect the endogenous MT in apples (Fig. S3). However, exogenous MT application significantly suppressed ethylene production in apples (Fig. 1F). Therefore, it can be inferred that MT plays a role upstream of ethylene and delays the senescence of apples by suppressing ethylene production. Nevertheless, this hypothesis requires further confirmation in mutants with MT deficiency and MT overaccumulation. A model for the suppression of ethylene synthesis by TF MdREM10 during fruit ripening was generated (Fig. 7). The expression of *MdREM10*, a key activator of ethylene biosynthesis, was markedly suppressed by MT treatment. MT inhibited MdREM10 from enhancing *MdERF3* and *MdZF32* transcription, thereby reducing *MdACS1* and *MdACO1* transcription and subsequently suppressing ethylene biosynthesis in apples. This indicated that MT mainly modulates ethylene biosynthesis by affecting the ethylene signal transduction pathway, rather than directly controlling the transcription of ethylene biosynthesis genes.

In addition to TFs in the ethylene signal transduction pathway, other TFs such as NAC, DOF, HSF, MYB, HD-Zip homeobox, and MADS-box proteins are also implicated in regulating fruit ripening [20]. These observations highlight the intricate regulatory networks orchestrated by TFs during fruit ripening. In this study, two new TFs, MdREM10 and MdZF32, were identified to be involved in ethylene biosynthesis and fruit ripening. B3 domain proteins form a significant superfamily of TFs characterized by B3 functional domains exclusive to plants [47]. They comprise several gene subfamilies, IAA response factor (ARF), abscisic acid-insensitive3 (ABI3), high-level expression of sugar inducible, related to ABI3/VP1 (RAV), and REM [48]. The REM family is the most diverse member of the B3 superfamily, yet its regulatory functions and molecular mechanisms are largely unknown [49, 50]. It has been postulated that REM may be involved in flower development [50] as well as in the development of female and male reproductive organs [49, 51]. The biological functions and molecular mechanisms of REM in apples are not yet understood. In this study, MdREM10, a member of the REM subfamily homologous to PbREM10 (XM_009342539.3), was identified in apples that exhibit a significant response to MT treatment. Our findings confirmed the crucial function of MdREM10 in the inhibition of ethylene synthesis by MT. Ethylene production in *MdREM10*-OE fruits was markedly elevated, resulting in accelerated ripening. The maturation process was advanced and showed no significant response to MT inhibition compared to the control group. These findings demonstrated that MdREM10 functions as a mediator between MT and ethylene. Nevertheless, further investigations must ascertain whether MdREM10 is necessary for the interaction between MT and other phytohormones. Prior research has demonstrated that the REM family lacks specific DNA-binding sequences [48]. Nevertheless, we confirmed that the core binding element sequence of MdREM10 is AAA(T/G)(G/T) using EMSA and ChIP-PCR assays. Furthermore, our findings revealed that MdREM10 activates the transcription of *MdERF3* and *MdZF32* by binding to specific sites on their promoters. These findings provided a foundation for further research into the regulatory role of REM as a TF.

ZFPs are also found in eukaryotes. The classification of ZFP is based on the number and order of conserved residues in the zinc finger domain, cysteine (C) and histone (H). The following ZFP types have been identified: C2H2, C3H, C2HC, C3HC4, C2HC5, C4HC3, C4, C6, and C8 [52]. Most CCCH proteins influence plant growth, development, and adaptive responses via hormonal action. PvC3H69 has been shown to suppress leaf senescence in switchgrass [53], while the CCCH protein SWOLLEN ANTHER WALL 1 (SAW1) positively regulates anther wall development via the gibberellins (GAs) signaling pathway in rice [54]. Recent genetic studies have elucidated the role of CCCH in hormone-mediated growth, environmental responses, and the regulation of gene expression in plants. Nevertheless, the precise molecular mechanisms underlying ZFP-mediated ethylene synthesis in apples remain unclear. In this study, *MdZF32* was cloned from GD fruits, and its subcellular localization was determined to be cytoplasmic and nuclear (Fig. 4B). MdZF32 suppressed ethylene biosynthesis by directly binding to the *MdACO1* promoter. Previous studies have demonstrated that CCCH proteins in rice and *Arabidopsis* are implicated in DNA binding, RNA binding, and mRNA turnover or silencing [55, 56]. Our findings revealed that MdZF32 binds to the *MdACO1* promoter and activates its transcription. Pomeranz *et al.* [56] showed that CCCH proteins can move between the nucleus and cytoplasm in response to different stressors. Nevertheless, further investigations must ascertain whether MdZF32 undergoes nucleocytoplasmic shuttling in response to MT treatment. The findings of this study provided compelling evidence that CCCH proteins play a direct role in ethylene biosynthesis.

This study revealed that the transcription of three TFs respond to MT treatment: MdREM10, MdZF32, and MdERF3. MdERF3 has been demonstrated to function as an activator of ethylene biosynthesis [13]. To elucidate the relationship between these factors in the context of MT-mediated inhibition of ethylene biosynthesis, we transiently overexpressed *MdREM10* (*MdREM10*-OE) and *MdZF32* (*MdZF32*-OE) in apples. *MdERF3* and *MdZF32* expression levels were significantly higher in *MdREM10*-OE fruits, while *MdREM10* and *MdERF3* expression levels remained unchanged in *MdZF32*-OE fruits. These results indicated that MdREM10 may be situated upstream of *MdZF32* and *MdERF3*. The MT treatment did not alter the expression levels of *MdERF3* and *MdZF32* in *MdREM10*-OE fruits. This indicated that the transcription of *MdERF3* and *MdZF32* necessitates the function of MdREM10 in response to MT treatment. Our findings demonstrated that MdREM10 binds to the promoters of *MdZF32* and *MdERF3*, whereas MdZF32 does not bind to *MdREM10* or *MdERF3*, as revealed by Y1H analysis. The binding sites of MdREM10 on the *MdZF32* and *MdERF3* promoters were identified both *in vitro* and *in vivo* using EMSA and ChIP-PCR analyses. In addition, its analysis demonstrated that MdREM10 promotes the transcription of *MdZF32* and *MdERF3*. In conclusion, MdREM10 functions as an upstream regulator of *MdZF32* and *MdERF3* expression. The MT-mediated repression of ethylene biosynthesis is achieved by regulating the transcription of downstream genes through MdREM10.

## Materials and methods

### Plant material and treatments

Apple (*M. domestica*, cv. ‘Golden Delicious’ (GD)) fruits were harvested at 140 DAFB from the Liaoning Pomology Institute orchard (Xiongyue, China) when the total soluble solid content reached 12%. Fruits were treated with MT by immersion in solutions of 0.01, 0.1, 0.5, 1, or 2 mM (Cat. no. M8600; Solarbio, Beijing, China) for 2 h. Ethephon (ethylene releaser) treatment was performed as described previously [57]. Control samples consisted of untreated fruits. Post-treatment, fruits were stored at 25°C for 20 days, during which samples were collected. *Nicotiana benthamiana* plants were cultivated in our laboratory following the method outlined by Li *et al.* [13].

### Determinations of MT and ethylene production in apple

MT was extracted and quantified, as described by Xu *et al.* [34]. The main extraction procedures involved an initial extraction with methanol, evaporation of the extraction solution to dryness, and using a C18 solid-phase extraction cartridge purifying extract (ProElut; Dikma, China). The measurement of MT content in apples was conducted using a UHPLC–MS system with an ACQUITY UHPLC system (Waters, Milford, MA, USA), following the methodology outlined by Ma *et al.* [23]. Ethylene production was measured as described previously [58].

### Reverse transcription-quantitative PCR

Total RNA extraction and cDNA synthesis were performed following the protocol outlined by Li *et al.* [13]. Gene expression was measured using RT-qPCR [59]. *MdActin* served as the internal reference gene. Primer sequences are provided in Supplemental Table 3.

### *Agrobacterium tumefaciens* infiltration

The CDS of *MdREM10* or *MdZF32* was cloned into the pRI101 vector with a GFP tag using *Kpn*I and *Bam*HI restriction sites, resulting in the constructs *Pro35S:MdREM10-GFP* or *Pro35S:MdZF32-GFP*. Plasmids were inserted into the *A. tumefaciens* strain (EHA105) via the heat-shock technique [13]. An infiltration assay was performed on the apples at the commercial harvest stage. A 1-ml sterile syringe was used to inject 1 ml of the suspension into fruits at a depth of 0.6 cm, with each fruit receiving 5-6 injections, and then stored at 25°C for 10 days. Samples were taken every 5 days, mainly from the pulp of the injection point and the surrounding area. Apple fruits injected with pRI101-GFP carrier were used as controls.

### Subcellular localization

*Pro35S:MdREM10-GFP* and *Pro35S:MdZF32-GFP* constructs were produced as described above. *Nicotiana benthamiana* leaves were injected with *Pro35S:MdREM10-GFP* or *Pro35S:MdZF32-GFP* injection buffer, using an empty GFP vector as a control. The mCherry-labeled NF-YA4-mCherry served as a nuclear marker [60]. Fluorescence was detected after 3 days using a confocal microscope (TCS SP8; Leica Wetzlar, Germany) as outlined in [14]. The representative results were presented after being repeated a minimum of three times.

### Yeast one-hybrid and yeast two-hybrid assays

The MdREM10 (254 aa), MdZF32 (580 aa), and MdERF3 (277aa) sequences were introduced into the pGADT7 vector. The MdREM10 (254 aa) and MdZF32 (580 aa) sequences were inserted into the pGBKT7 vector. Promoter fragments of *MdREM10*, *MdZF32*, *MdERF3*, *MdACO1*, and *MdACS1* were each cloned into the pAbAi vector. The Y1H and Y2H assays were conducted according to the method described by Ji *et al.* [14]. Supplemental Table 3 lists all primers used.

### EMSA

The *MdZF32* CDS was inserted into pET-30a^+^ (TaKaRa, Kyoto, Japan) to produce MdZF32-His. The *MdREM10* coding sequence was inserted into the pGEX4T-1 vector (GE Healthcare, Chicago, IL, USA) to produce the MdREM10-GST construct. Competent *Escherichia coli* BL21 (DE3) cells were transfected with the plasmids. The recombinant fusion proteins were purified using the method outlined by Li *et al.* [13]. A biotin-labeled double-stranded DNA probe was prepared for EMSA, with the label attached at the 3′ end. The EMSA was performed using a Light Shift Chemiluminescence EMSA Kit (Beyotime, catalog no. GS009, Shanghai, China), following the method outlined in [14]. Supplemental Table 3 lists all primers used.

### *β*-Glucuronidase analysis

Promoter fragments of *MdZF32* (2000 bp), *MdACO1* (2000 bp), and *MdERF3* (1162 bp) were inserted upstream of the GUS reporter in the pCAMBIA1301 vector (Novagen, Madison, WI), replacing the *35S* promoter via *Hind*III and *Nco*I restriction sites. *MdREM10*, also known as *MdZF32*, was incorporated into the pRI101 vector to serve as an effector. The reporter and effector vectors were introduced into EHA105 cells and simultaneously infiltrated into *N. benthamiana* leaves. The coinfiltration and examination of the GUS activity followed the method outlined by Li *et al.* [13].

### Dual-luciferase reporter assay

The *MdZF32*, *MdACO1*, or *MdERF3* promoter sequences were cloned into the pGreenII0800-Luc vector to create the reporter construct. The effector construct was created by inserting the *MdREM10* or *MdZF32* CDS into the pGreenII62-SK vector. The transformation of reporter and effector constructs into *N. benthamiana* leaves was conducted according to the method described by Li *et al.* [20].

### Chromatin immunoprecipitation-PCR analysis

The recombinant *Pro35S:MdREM10-GFP* and *Pro35S:MdZF32-GFP* constructs were transformed into apples. ChIP assays were conducted following established protocols [61] with anti-GFP antibodies (Transgen, Beijing, China). The quantity of immunoprecipitated chromatin was measured using qPCR, following the method outlined by Ji *et al.* [27]. Supplemental Table 3 lists the primers used.

### RNA-seq of apple fruits

Control and MT-treated apples sampled 10 days after storage (harvested at 140 DAFB) were used for RNA-seq analysis. cDNA library construction, sequencing, and bioinformatic analyses were performed as previously described [62]. RNA sequencing was performed using an Illumina NovaSeq6000 by Biomarker (http://www.biomarker.com.cn/). Relevant data are available in the manuscript and supporting material. Study data can be accessed in the NCBI database under accession number PRJNA1150269.

## Supplementary Material

Web_Material_uhaf020

## Data Availability

The sequence data utilized in this study can be accessed through the Genome Database for Rosaceae (https://www.rosaceae.org) and the apple genome repository at the National Center for Biotechnology Information (NCBI). The sequence accession numbers are: *MdREM10* (MD03G1230200), *MdZF32* (MD03G1280300), *MdERF2* (AB288348), *MdERF3* (XM_008339725), *MdERF4* (MD03G1231800), *MdERF5* (XM008365562), *MdACS1* (U89156), *MdACO1* (AF030859), *MdCOMT1* (XM029104846), *MdCOMT2* (NM001434474), and *MdActin* (EB136338).
