# Supplementary material for: Melatonin suppresses ethylene biosynthesis by inhibiting transcription factor MdREM10 during apple fruit ripening
Source: Hortic Res. 2025 Jan 21;12(5):uhaf020. doi: 10.1093/hr/uhaf020 (PMC11975395; doi:10.1093/hr/uhaf020)
Supplement: Web_Material_uhaf020 [file web_material_uhaf020.zip › Supplementary Figure.pdf]

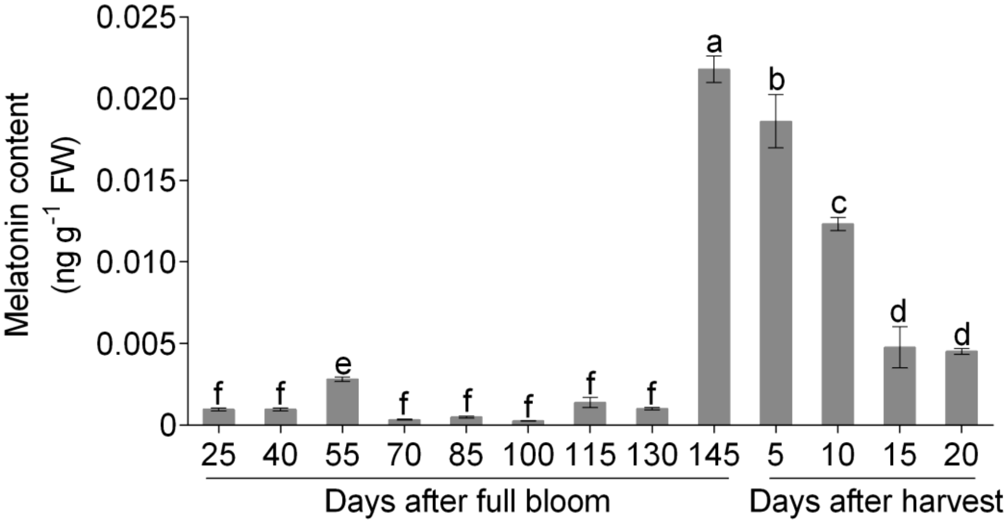

Supplemental Figure 1. Endogenous melatonin (MT) levels during apple fruit development and ripening in 2021.

Fruits collected at 25, 40, 55, 70, 85, 100, 115, 130 and 140 DAFB in 2021. The commercial harvest date of GD fruits is 140 DAFB, and fruits stored at 25°C at this time for 20 days. MT content was measured. Values are presented as means ± SE. Different lowercase letters indicate a significant difference at *P<0.05* based on one-way ANOVA test.

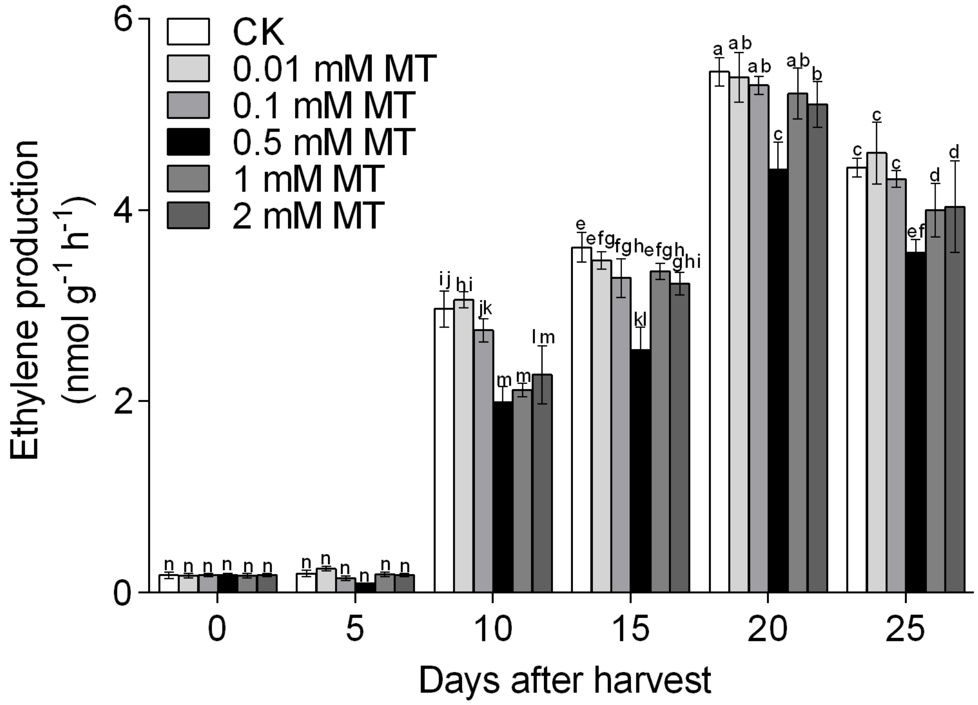

Supplemental Figure 2. The Ethylene production in apple fruits treated with different concentrations of melatonin (MT).

Fruits collected on the day of commercial harvest (140 DAFB) in 2021 were treated with MT (0.01, 0.1, 0.5, 1, or 2 mM) and stored at 25°C for 20 days. Ethylene production was measured after treatment. MT, the apple fruits were treated with MT; CK, the apple fruits were not treated as the control. Values are presented as means ± SE. Different lowercase letters indicate a significant difference at *$P<0.05$* based on two-way ANOVA test.

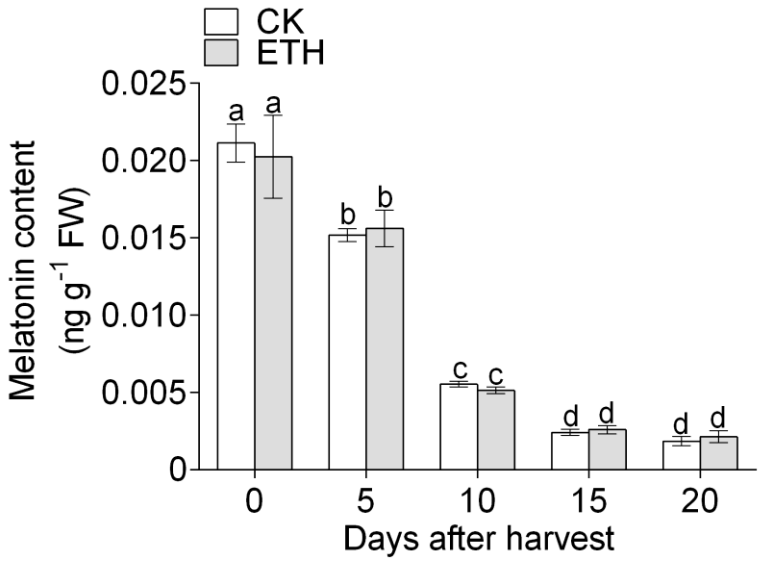

Supplemental Figure 3. The melatonin (MT) content in apple fruits treated with ethephon.

Fruits collected on the day of commercial harvest (140 DAFB) in 2022 were treated with ethephon and stored at 25°C for 20 days. Ethylene production was measured after treatment. ETH, the apple fruits were treated with ethephon; CK, the apple fruits without treated as the controls. Values are presented as means ± SE. Different lowercase letters indicate a significant difference at *P<0.05* based on two-way ANOVA test.

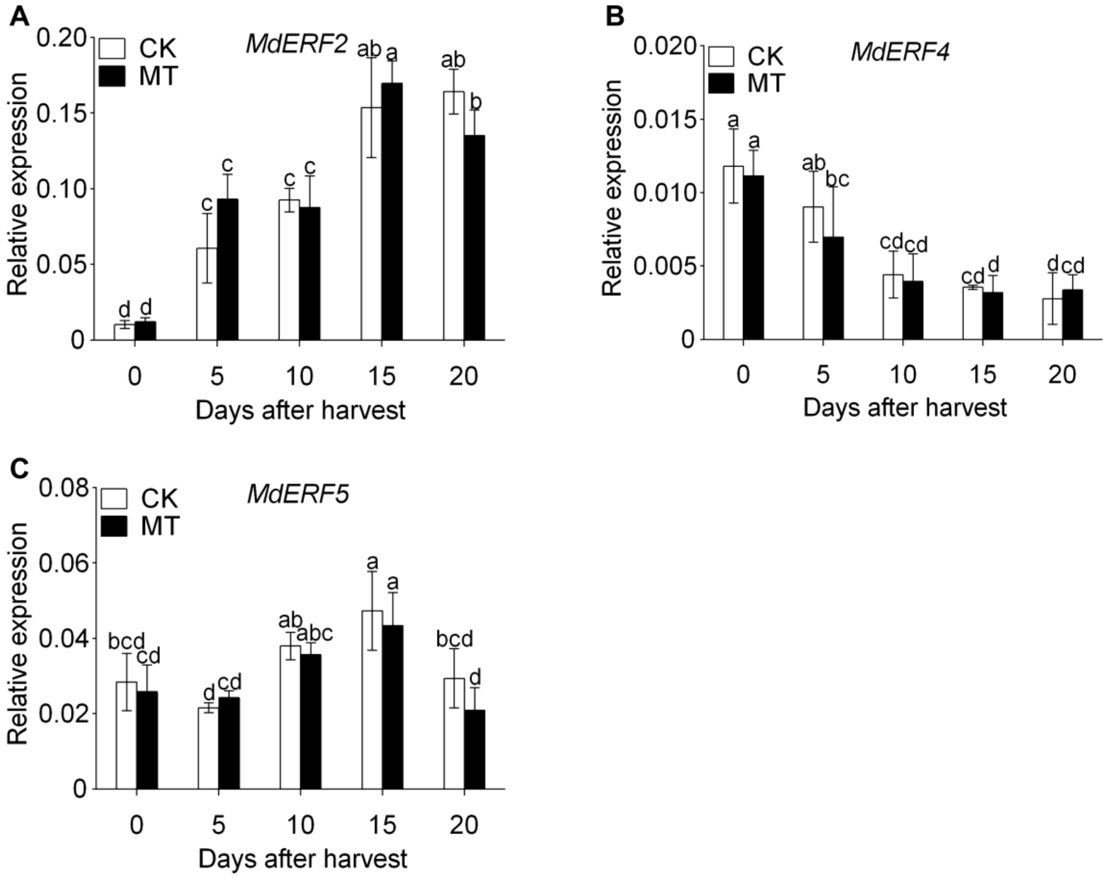

Supplemental Figure 4. Expression of *MdERF2*, *MdERF4* and *MdERF5* in apple fruits treated with melatonin (MT).

RT-qPCR was used to evaluate the expressions of *MdERF2*, *MdERF4* and *MdERF5* in apple fruits treated with MT. The X-axis represents the number of days stored at 25°C after harvest. A total of three biological replicates were analyzed. Values are presented as means ± SE. Different lowercase letters indicate a significant difference at *P<0.05* based on two-way ANOVA test.

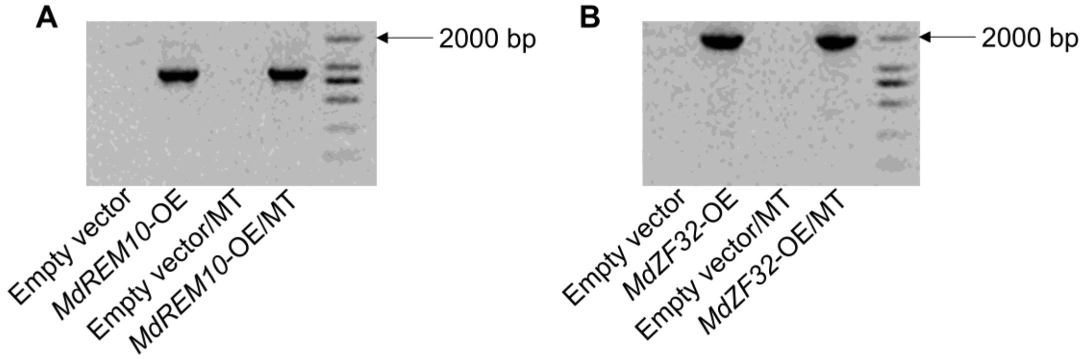

Supplemental Figure 5. Detection of *MdREM10* and *MdZF32* overexpression at DNA level.

Agrobacterium-mediated transient overexpression of *MdREM10* (*MdREM10*-OE) and *MdZF32* (*MdZF32*-OE) in apple fruits harvested at 140 days after full bloom. Infiltrated fruits were confirmed by PCR amplification with the primers (5′-GACGCACAATCCCACTATCC-3′ and 5′-CTAAAAAATCATGGTGTCCCCG-3′) and (5′-GACGCACAATCCCACTATCC-3′ and 5′-TTAAATCGATCGACGAAACGC-3′). The results showed that there was a clear band in the overexpressed apple fruits, but no corresponding bands in the controls, indicating that the overexpression was successful.

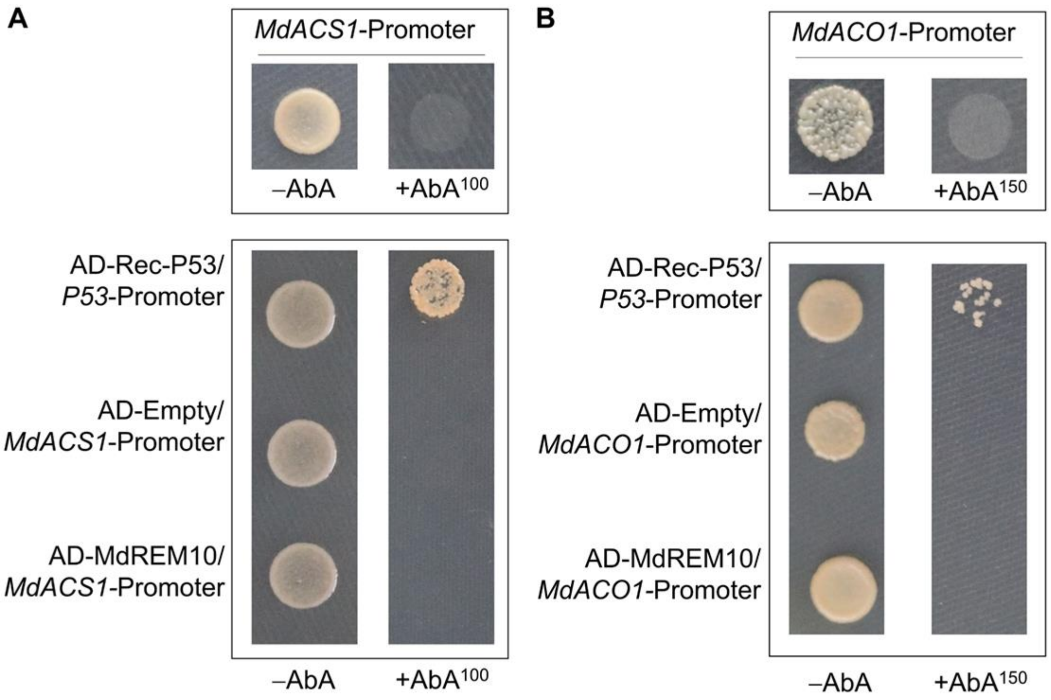

Supplemental Figure 6. MdREM10 does not bind to the *MdACS1* and *MdACO1* promoters.

Y1H analyses revealed that MdREM10 did not bind to the promoter of *MdACS1* and *MdACO1*. AD-Rec-P53/*P53*-Promoter acted as a positive control. AD-Empty/*MdACS1*-Promoter or AD-Empty/*MdACO1*-Promoter worked as a negative control.

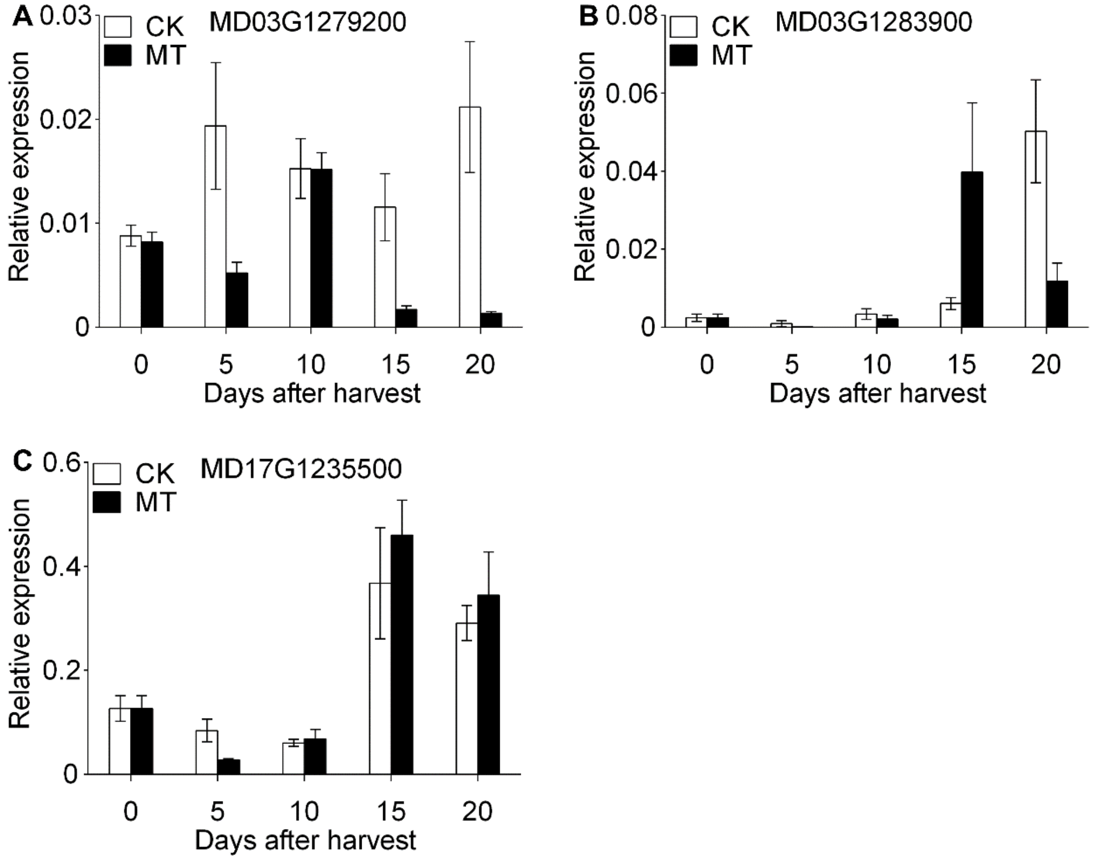

Supplemental Figure 7. Expression of three genes coding zinc finger proteins in apple fruits treated with melatonin (MT).

RT-qPCR was used to detect the expression of MD03G1279200, MD03G1283900 and MD17G1235500 in apple fruits treated with MT. The X-axis represents the number of days stored at 25°C after harvest. A total of three biological replicates were analyzed. Values are presented as means ± SE.

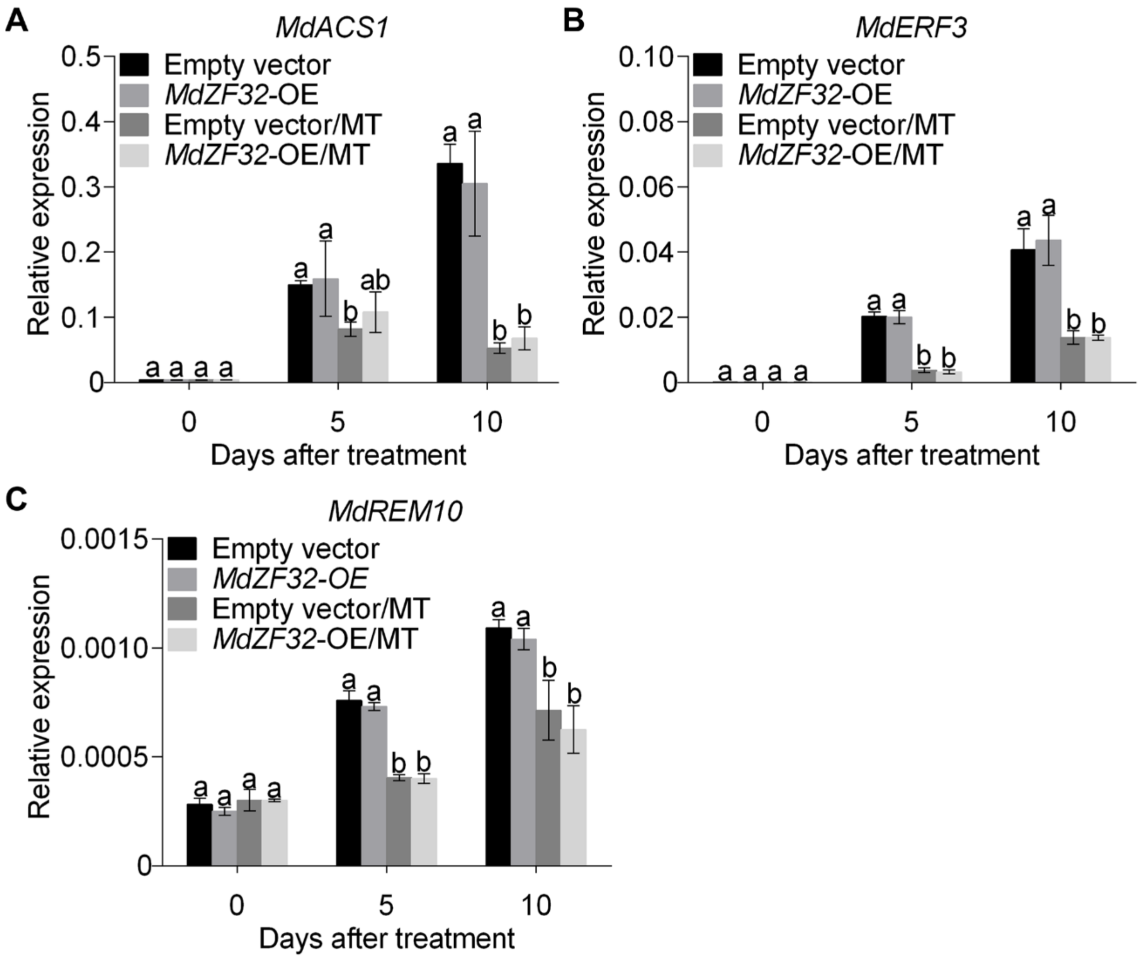

Supplemental Figure 8. The genes expression in *MdZF32*-OE fruits.

Overexpression of MdZF32 (*MdZF32*-OE) in apple fruits harvested at 140 DAFB using *Agrobacterium*-mediated transient transformation. 24 h after injecting, immediately treated with MT and stored at 25°C for 10 days. Fruits infiltrated with empty pRI101-GFP vectors were used as controls. RT-qPCR was used to evaluate the *MdACS1*, *MdERF3* and *MdREM10* expression in *MdZF32*-OE fruits. Values are presented as means ± SE. Different lowercase letters indicate a significant difference at $P<0.05$ based on one-way ANOVA test.

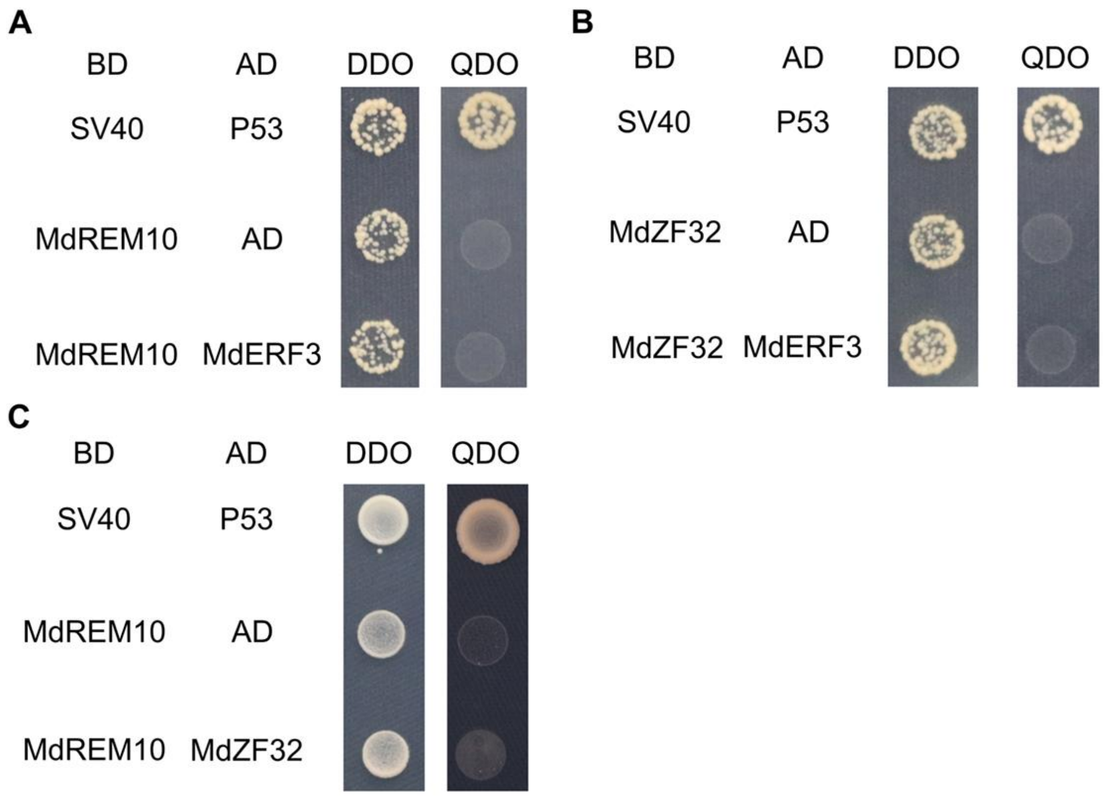

Supplemental Figure 9. Yeast two-hybrid analyses of protein interactions.

Y2H analyses revealed that MdERF3 was not interacted with MdREM10 or MdZF32 and MdREM10 does not interact with MdZF32. DDO, SD medium lacking Trp and Leu; QDO, SD medium lacking Trp, Leu, His, and Ade. SV40-BD and P53-AD were used as a positive control. MdREM10-BD and AD, MdZF32-BD and AD were respectively used as negative controls.

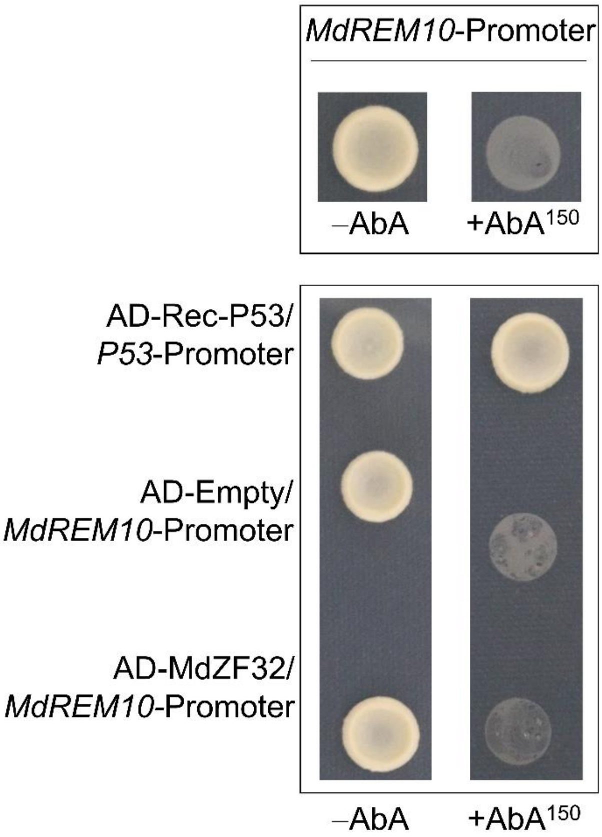

Supplemental Figure 10. MdZF32 does not bind to the *MdREM10* promoter.

Y1H analysis revealed that MdZF32 did not bind to the promoter of *MdREM10*. AD-Rec-P53/*P53*-Promoter acted as a positive control. AD-Empty/*MdREM10*-Promoter worked as a negative control.
